# ParentWorks: Evaluation of an Online, Father-Inclusive, Universal Parenting Intervention to Reduce Child Conduct Problems

**DOI:** 10.1007/s10578-019-00934-0

**Published:** 2019-10-24

**Authors:** Patrycja J. Piotrowska, Lucy A. Tully, Daniel A. J. Collins, Vilas Sawrikar, David Hawes, Eva R. Kimonis, Rhoshel K. Lenroot, Caroline Moul, Vicki Anderson, Paul J. Frick, Mark R. Dadds

**Affiliations:** 1 School of Psychology, University of Sydney, Sydney, NSW 2006 Australia; 2 School of Psychology, University of New South Wales, Kensington, NSW 2052 Australia; 3 School of Psychiatry, Faculty of Medicine, University of New South Wales, Kensington, NSW 2052 Australia; 4 Royal Children’s Hospital, Murdoch Children’s Research Institute & Departments of Psychology & Paediatrics, University of Melbourne, Parkville Campus, Parkville, VIC 3010 Australia; 5 Department of Psychology, Louisiana State University, Baton Rouge, LA 70803 USA; 6 Learning Sciences Institute of Australia, Australian Catholic University, Banyo, QLD 3010 Australia

**Keywords:** Online parenting interventions, Parenting, Fathers, Child behavioural problems

## Abstract

Evidence-based parenting interventions are effective in reducing conduct problems, yet these interventions have limited reach, and few involve the participation of fathers. This paper describes the outcomes of an open trial of *ParentWorks,* a universal, online, father-inclusive parenting intervention aiming to decrease childhood behavioural problems and promote positive parenting in mothers and fathers. A total of 388 families (456 individual parents; 36.6% fathers) were included in the study. Mixed model analyses showed significant decreases in child emotional/behavioural problems, dysfunctional parenting, interparental conflict, and parental mental health problems. The baseline severity of child behavioural problems significantly moderated the effects on child outcomes so that children with higher levels of problems benefitted more from the program. Participation of both caregivers in two-parent families, as well as parent sex, did not significantly affect the program outcomes. Results provide initial empirical support for the universal, self-directed, online parenting intervention, in addressing both child behavioural problems and parenting outcomes. *Trial registration: ACTRN12616001223426, registered 05/09/2016.*

**Electronic supplementary material:**

The online version of this article (10.1007/s10578-019-00934-0) contains supplementary material, which is available to authorized users.

## Introduction

Child mental health problems pose an important challenge to many societies, and remain a critical target of social policies given the lifetime burden and costs for individuals, families and the community. A recent meta-analysis of 41 studies from 27 countries estimated the prevalence of mental disorders in children and adolescents at 13.4%, indicating that a significant number of children worldwide experience mental health problems [1]. More than one-third of these children are diagnosed with disruptive behaviour disorders including oppositional defiant disorder (ODD) and conduct disorder (CD), which are more broadly referred to as behavioural or conduct problems and range from persistent rule-breaking through to physical aggression [2]. Conduct problems, especially in their most disruptive forms, may activate a chain of negative cascading effects leading to long-term adverse outcomes such as lower academic achievement, mental health problems, involvement in the criminal justice system and substance dependence [3–6], leading to significant societal financial burden [7].

Child mental health problems such as conduct problems can be effectively addressed by early parenting interventions. These programs, also referred to as ‘parenting programs’ or ‘parent training’, target parenting skills and the quality of parent–child relationships in order to improve child behavioural outcomes. A substantial evidence base suggests that parenting programs based on social learning and cognitive behaviour theories are effective in reducing child mental health problems including conduct problems [8, 9]. Several meta-analytic reviews have found that these programs can also improve a range of psychosocial outcomes for parents such as mental health [10, 11] and satisfaction with the partner relationship [10]. Despite their effectiveness, only a minority of parents of children and adolescents with mental health problems participate in these programs [12]. Some of the most significant barriers to intervention participation include financial cost, practical factors (e.g., location, transport), waiting times, and social and personal stigma associated with child mental health problems and help-seeking [13]. Consequently, online delivery has become one of the ways to increase program reach, and reduce stigma as well as meet the practical demands of participation for families.

Previous systematic reviews have demonstrated that online parenting interventions improve child behaviour, parental confidence, and dysfunctional parenting, with effect sizes similar to face-to-face interventions [14, 15]. Most online parenting interventions delivered to date, however, have included some practitioner involvement, and there is a need to examine the effectiveness of online interventions that are entirely self-directed. Such interventions have the greatest potential for population reach, and are likely to be cost-effective as ongoing delivery costs are minimal.

Regardless of delivery modality, however, fathers are consistently underrepresented in parenting interventions and research. The majority of parenting intervention studies do not report rates of father participation [16] and, when rates are reported, they remain low. For example, a review of 28 studies on parenting interventions found only 20% of parents enrolled in parenting interventions were fathers [17]. This is especially significant given research highlighting the importance of participation of both parents—the ‘parenting team’ [18]—and a meta-analysis showing that programs that included fathers reported significantly more positive changes in children’s behaviours and better parenting practices following the intervention [19]. Research also suggests that parenting interventions may be less effective for fathers than mothers [17]; it is therefore important to examine intervention outcomes separately based on parent sex. Research on online interventions has primarily focused on mothers and, to the best of our knowledge, no evaluations have examined outcomes for the participation of two versus one parent. Online interventions may address some of the practical barriers to father participation such as cost of service, lack and time and work commitments, and survey research has found that fathers prefer online over face-to-face interventions [20, 21]. Thus, online formats may be particularly well-suited to fathers, and ensure high rates of father engagement.

This study examined the outcomes of an open trial of a universal, self-directed, online, father-inclusive parenting program, called *ParentWorks,* an adaptation of an existing evidence-based parenting program [22]. The intervention, and an associated media campaign, were rolled out across Australia throughout 2016 as the first freely available, online parenting program for child conduct problems. Evaluating universal online mental health programs is challenging as it is difficult to randomize families to receive the intervention versus a waitlist or control, especially when there is no therapist involvement in the program. Thus, as a first step in evaluating the impact of the program in the real world, we examined pre- and post-intervention scores on child and family measures of mental health. In addition, for the group who reported high levels of child behaviour problems at pre-intervention, we compared their outcomes to a comparison group of families who had previously participated in face-to-face and telehealth (therapist sessions delivered via teleconferencing) versions of the same intervention [23, 24]. This benchmarking analysis was done to examine the effects of an entirely self-directed online intervention to the same intervention with differing degrees of therapist involvement. It should be noted that this study does not examine drop-out rates since an earlier study on a smaller sample of ParentWorks participants examined and reported on rates of drop-out and explored a range of demographic, parent and child variables as predictors of drop out [25].Given the focus on being father-inclusive, the study also examined the differential impact of participation of two versus one parent (in two-parent families). Based on previous research [19], we expected that participation of two parents would result in significantly greater improvements in child behavioural and parenting outcomes following the intervention. Previous research on face-to-face interventions has also demonstrated that greater severity of child behaviour problems and younger child age are associated with larger positive effects on child outcomes [26, 27], and some research suggests that parental mental health may affect parent outcomes [28]. Consequently, in addition to parent participation status, the baseline severity of child behaviour problems, child age, and parental mental health were included in analyses as potential moderators of intervention outcomes. Given that self-directed online parenting interventions have potential for significant cost-effectiveness, this study also examined the program’s costs relative to benefits. This study is reported in accordance with the TREND statement for the reporting of intervention evaluation studies with non-randomized designs [29].

## Methods

For the full sample outcomes, this study was a single group clinical trial involving a quasi-experimental, repeated-measures design with three assessments (pre-intervention, post-intervention and three-month follow-up). Results are reported here for pre- and post-intervention only, as collection of follow-up data was ongoing at the time of writing this paper. Full study details have been described in the study protocol [30]. The trial was registered with the Australian New Zealand Clinical Trials Registry (ACTRN12616001223426), and was approved by the University of Sydney Human Research Ethics Committee (Project No. 2016/452). For the subsample reporting high levels of child behaviour problems at pre-intervention, the design was a three group non-randomized benchmarking comparison at pre- and post-intervention (*Parentworks*, face-to-face, and telehealth treatment).

### Participants and Recruitment

A national media campaign was conducted online and through social media, to directly target the involvement of fathers in *ParentWorks* [31]. Participants (mothers and fathers) were also recruited through word of mouth, flyers available in child and family services, or practitioner recommendation. Interested participants were directed to the program website to access information and register for the program. Inclusion criteria were: parent or caregiver (over 18 years old) of a child aged 2–16 years, currently living in Australia, and able to complete questionnaires and understand program content in English. This study included parents who completed the program between August 2016 and January 2019.

### Intervention

*ParentWorks* was based on a face-to-face intervention for parents of children with conduct problems [22], for which effectiveness in a telehealth web-based format was previously reported [24]. The intervention was modified to be suitable for a broader community sample of parents, including those with more general concerns about parenting and child behaviour. The program comprised video presentations of six interactive sequenced ‘modules’, five of which were compulsory. The modules included topics such as encouraging positive behaviours, responding to misbehaviours, and working as a team; each module took approximately 20–30 min to complete. Full details of the intervention are described elsewhere [30]. The core principles of the intervention are, like many evidence-based parenting interventions, appropriate for parents of all children aged 2–16 years; however, because the specifics of certain parenting strategies vary by child age (e.g., use of time-out and other discipline strategies), the intervention provides an additional tipsheet for managing misbehaviour in older children and teenagers.

### Measures

At registration, participants answered sociodemographic questions about themselves and their families, and entered information about the ‘target’ child, that is, the child aged 2–16 years whose behaviour or development was concerning the parent/caregiver (or if no concerns, their youngest child aged 2–16). The primary outcomes—child total emotional/behavioural problems, child conduct problems, dysfunctional parenting, interparental conflict, and parental mental health—were measured at pre-and post-intervention by all participants (both mothers and fathers). Satisfaction with the program was measured post-intervention.

Participants answered questions about their age, sex, marital status, education level and whether English was the primary language spoken at home. They also reported the number of children, the target child’s age and sex, whether they had ever sought help from a health practitioner for their child’s emotional/behavioural difficulties and, if so, whether the child had been diagnosed with any disorder. All participants were asked if they were completing the program alone or with someone else.

*Child behavioural difficulties* were measured using the Strengths and Difficulties Questionnaire [SDQ; [32] ]. The SDQ is a 25-item parent-report questionnaire which measures child emotional and behavioural adjustment. Each item is rated on a 3-point scale from 0 (not true) to 2 (certainly true). The Total Difficulties (SDQ-T) score (*α *= 0.79–0.86) was used to measure total emotional/behavioural problems (range 0 to 40) and the Conduct Problems (SDQ-CP) subscale (*α *= 0.72–0.80) was used to measure conduct problems (range 0 to 10).

*Parental mental health* was assessed with the Kessler Psychological Distress Scale [K6; [33, 34]]. The K6 is a 6-item self-report questionnaire which asks about anxiety and depression symptoms in the previous 4-week period (*α *= 0.86). Respondents rate each question from 1 (none of the time) to 5 (all of the time); higher sum scores indicate higher levels of psychological distress (range 6 to 30).

*Dysfunctional parenting* was assessed using the Parenting Scale of the Parenting and Family Adjustment Scales [PAFAS; [35]]. The Parenting scale contains 18 items assessing both dysfunctional and positive parenting (*α *= 0.81). Parents rate each item on a scale from 0 (not true of me at all) to 3 (true of me very much, or most of the time), with higher scores indicating higher levels of dysfunctional parenting (range 0 to 54).

*Interparental conflict* over parenting was assessed in two-parent families (parents in married and defacto relationships) using the Parent Problem Checklist [PPC; [36]]. The PPC is a 16-item self-report questionnaire measuring conflict between parents specifically relating to child-rearing practices, e.g., disagreements over type of discipline or sharing childcare workload. For each item, parents report whether or not the issue has been a problem over the last 4 weeks. Scores range from 0 to 16, with higher scores indicating greater conflict over child-rearing (*α *= 0.85).

*Satisfaction* with the program was assessed at post-intervention with five items from the Client Satisfaction Questionnaire [37] adapted from the Therapy Attitude Inventory [38]. The items (rated on a 7-point Likert scale) asked about parents’ satisfaction with the program, whether it helped them deal more effectively with their child’s behaviour and other family problems, as well as whether their child’s behaviour and/or the relationship between parents improved (the latter question asked of married/de facto participants only).

### Procedure

Parents enrolling in *ParentWorks* were required to read the online participant information statement and indicate their consent to participate. Information from participating parents was collected through online questionnaires. Before starting the program, participants completed sociodemographic questions and pre-intervention measures. They could then access the first two modules immediately. Each of the remaining modules was unlocked 1 week after the previous module. After completing the final compulsory module, parents completed post-intervention measures. Parents had to complete the five core modules in order to complete post-intervention measures. No identifying information was included in the dataset.

### Statistical Analyses

Dropout data analyses were conducted on a subsample of participants who fully registered for *ParentWorks*; details are reported elsewhere [25]. In the current sample, 4315 (90.4%) were considered drop-outs. Given the amount of missing data, imputation methods and intent-to-treat analyses were considered not feasible and the current study focused on individual parents who completed *ParentWorks*. The data were first inspected and sample described using descriptive statistics and frequencies. To examine intervention outcomes, a series of mixed-model repeated-measures analyses of variance (ANOVAs) were conducted on all outcomes (for 68 children, the data were available from two parents and thus non-independent, therefore the following analyses were re-run excluding the second caregiver; results did not substantially differ from those presented here, and are available upon request). Time (from pre- to post-intervention) was entered as a within-subject factor in all models. The first set of ANOVAs examining child outcomes (SDQ-T and SDQ-CP) included child age and parent sex as between-subject factors, as well as pre-intervention severity of child behavioural symptoms (SDQ-T-cat and SDQ-CP-cat respectively). For these latter variables, SDQ scores were categorized into four groups according to symptom severity: close to average, slightly raised, high, and very high [39]. The ANOVAs of parent outcomes (K6, PAFAS, PPC) included parent sex and parental mental health (K6) entered as between-subject factors. K6 was categorized to indicate likely mental health disorder on the basis of the clinical cut-off score of 19 (K6-cat). These models allowed the interactions between time and the between-subject factors on parent outcomes to be explored. All ANOVAs with the exception of PPC were conducted on the entire sample. As the PPC was only administered to married/de facto participants, single caregivers were excluded from this analysis.

The benchmarking analysis was conducted to evaluate intervention effects against a sample of children who had previously been referred and treated for conduct problems with the core parenting program provided in face–face or telehealth web-based formats [23, 24]. Comparisons with the benchmarking samples were limited to children rated as *high* or *very high* for conduct problems (face–face: *N* = 83; web-based: *N* = 87; ParentWorks: *N* = 151) and consisted of comparing intervention outcomes on SDQ Conduct Problems using repeated measures ANOVA. Due to variable participation from fathers across groups, benchmarking analysis was limited to mothers’ rating of SDQ Conduct Problems.

Prior to running the second set of models investigating the effect of two-caregiver participation on the outcomes, associations between participation status and pre-intervention measures were explored to determine whether there was any significant difference on the basis of whether parents chose to participate alone or with another caregiver. These analyses revealed a significant relationship with the initial level of emotional/behavioural problems. Consequently, all the following models included two between-subject factors: participation status and SDQ-T-cat. Interactions with time as well as between those two factors were allowed in the model. These models were only run for married/de facto participants since almost all single caregivers (97.8%) reported completing the program on their own. All main effects and interactions are presented in the results section. Significant interactions were further examined with simple main effects analyses with Bonferroni corrections.

Participants’ satisfaction with the program was explored using descriptive statistics, and potential differential effects for parent sex were examined using an independent samples *t* test. Finally, program cost-effectiveness was estimated by calculating the total and ongoing costs of developing and delivering *ParentWorks* and presenting it in the context of changes in child outcomes. In addition, we calculated the number of children who moved out of the clinical range on the SDQ-CP (pre- to post-intervention assessment) and the cost of disorder potentially averted. All analyses were conducted in IBM SPSS Statistics 22.

## Results

### Sample Descriptives

As previously mentioned, the sample comprised individual parents who completed all pre- and post-intervention assessments for the program. The total sample for the current study included 388 families (456 individual caregivers) of children ranging in age from 2 to 16 years; sample descriptives are presented in Table 1 (descriptive data were missing for a small number of participants due to website/data recording errors). Almost half of participating caregivers were single (49.8%) with the vast majority of these individuals completing the program on their own (97.8%). The remaining participants were married or in a de facto relationship; among them, 53.9% completed the program on their own and 46.1% completed with their partner (i.e., parenting team). Among married/de facto participants who completed the program on their own, the most frequently endorsed reasons for the second caregiver not participating were lack of time (45.0%) and the primary caregiver choosing to complete on his/her own (40.0%). Overall, 456 parents completed the program (62.9% female) with a mean age of 37.74 years. Overall, 36.6% of parents completing the program were fathers (41.08% when excluding single mothers). The majority of parents (95.2%) reported English as their main language and over half (52.9%) had a University degree. Approximately 40% of participants worked full time, and a quarter worked part-time (26.3%).

**Table 1:** Sample characteristics for total sample

Variable	Mean (SD) N (Valid %)
Family/child characteristics (N = 388)	
Family type^a^	N (%)
Single completing alone	185 (48.7%)
Single completing with someone else	4 (1.1%)
Married/de facto completing alone	103 (27.1%)
Married/de facto completing together	88 (23.2%)
Child	
Sex	
Female	154 (39.7%)
Male	234 (60.3%)
Previous help sought	175 (45.1%)
	Mean (SD)
Age	5.88 (3.49)
Parent characteristics (N = 456)^b^	
Sex	
Female	287 (62.9%)
Male	167 (36.6%)
English as the main language	434 (95.2%)
Education	
Primary/secondary school—year 10	86 (18.9%)
Secondary school—year 12/college	127 (27.9%)
University degree	241 (52.9%)
Employment	
Full-time	177 (38.8%)
Part-time	120 (26.3%)
Stay at home	95 (20.8%)
Unemployed	62 (13.6%)
	Mean (SD)
Age	37.74 (8.72)

^a^Based on first caregiver’s response to the participation question. In the final dataset, there were 68 parenting teams who completed the program together (at the time of data download)

^b^The sample includes 456 individual parents/caregivers; 2 missing data points for the descriptive statistics

The final sample included 388 children (60.3% boys), between 2 and 16 years (*M *= 5.88), with 57% under age 6. Just over 45% of families reported seeking previous help for their child’s behavioural/emotional problems; among those, the most commonly reported diagnoses were anxiety disorder (22.5%), ADHD (19.7%), developmental problems (19.7%), and ODD (10.4%).

### Satisfaction

The average satisfaction scores ranged from 2 to 7, with a mean of 5.49 (SD= 0.95), indicating participants were highly satisfied with the program. There were no significant sex differences in satisfaction ratings, *t*(452) = 0.41), *p *> 0.05, showing that fathers and mothers were equally satisfied with the program.

### Intervention Outcomes

Estimated means and standard errors for outcome measures at pre and post assessments are presented in Table 2. The first set of models for child outcomes showed significant main effects of time for both SDQ-T and SDQ-CP (see Table 3). Both child total emotional/behavioural and conduct problems outcome scores significantly decreased following the intervention. Importantly, models for both outcomes returned one significant interaction between time and initial severity of behavioural problems (SDQ-T-cat); the remaining two interactions were not significant (*p *> 0.05). The significant interaction suggests that the change in scores from pre- to post-intervention varied based on initial severity. Simple effects analyses showed significant improvements in child total behavioural outcomes for the three (out of four) most severe groups with mean pre-post differences between 3.24 and 6.10 for SDQ-T, and between 0.87 and 2.38 for SDQ-CP (all *p*’s< 0.01). Children with the lowest level of behavioural problems (‘close to average’) did not show significant changes from pre- to post-intervention on either outcome. There was no main effect of child age on either SDQ-T or SDQ-CP, with all three age groups presenting comparable scores (*p *> 0.05). Parent sex did not have a main effect on either of the two child outcomes, with similar scores reported by fathers and mothers (*p *> 0.05).

**Table 2:** Estimated means/SEs for all outcomes at pre and post assessments

	Mean (SE)	
	Pre	Post
SDQ total difficulties (SDQ-T)	15.93 (0.19)	12.81 (0.33)
SDQ conduct problems (SDQ-CP)	4.03 (0.06)	2.93 (0.11)
Parenting (PAFAS)	17.27 (0.61)	19.97 (0.56)
Interparental conflict (PPC)	5.93 (0.49)	3.84 (0.43)
Parental mental health (K6)	16.39 (0.32)	13.15 (0.33)

**Table 3:** Mixed-model results for child behavioural outcomes

Variables	SDQ total difficulties		SDQ conduct problems	
	F(df)	η_p_^2^	F(df)	η_p_^2^
Within-subject				
Time	94.32 (1,439)**	0.18	110.22 (1,445)**	0.20
Between-subject				
SDQ-cat (T or CP)	328.26 (3,439)**	0.69	415.7 (3,445)**	0.74
Child age	2.62 (2,439)	0.01	1.39 (2,445)	0.01
Parent sex	0.07 (1,439)	0.00	0.08 (1,445)	0.00
Interactions				
Time × SDQ-T-cat	40.6 (3,439)**	0.22	49.66 (3,445)**	0.25
Time × Child age	0.16 (2,439)	0.00	1.02 (2,445)	0.01
Time × Parent sex	0.25 (1,439)	0.00	0.33 (1,445)	0.00

*p < 0.05 **p < 0.01

The parent outcome models showed significant main effects of time (see Table 4) with all outcomes improving from pre- to post-intervention (all *p*’s< 0.01). Time effects interacted with K6-cat for parenting and parental mental health outcomes. Both caregivers mental health severity categories were associated with significant improvements in parenting, however, the mean difference for the less severe group was 2.71 (*p *< 0.01), and for the more severe group was 5.89 (*p *< 0.01); suggesting that parents with more serious mental health problems improved substantially more on parenting following the intervention. Likewise, caregivers in both severity categories improved significantly in their mental health; however, the mean difference for the less severe group was 0.81 (*p *< 0.01), and for the more severe group was 5.68 (*p *< 0.01); suggesting that parents with more serious mental health problems benefitted more from the intervention. The models also indicated that severity of initial mental health problems had a significant main effect on each parent outcome. Caregivers in the more severe mental health category generally reported higher levels of dysfunctional parenting (*M*_diff_= 5.71, *p *< 0.01), interparental conflict (*M*_diff_= 1.68, *p *< 0.05), and parental mental health (*M*_diff_= 9.08, *p *< 0.01). Parent sex did not have a main effect on parenting or parental mental health outcomes, with similar scores reported by fathers and mothers (*p *> 0.05).

**Table 4:** Mixed-model results for parent outcomes

Variables	Parenting (PAFAS)		Interparental conflict (PPC)		Parental mental health (K6)	
	*F* (1,451)	η_p_^2^	*F* (1,261)	η_p_^2^	*F* (1,451)	η_p_^2^
Within-subject						
Time	72.37**	0.14	26.77**	0.09	97.49**	0.18
Between-subject						
Parent sex	0.00	0.00	0.41	0.00	3.76	0.01
K6-cat	29.70**	0.06	4.16*	0.02	264.28**	0.37
Interactions						
Time × Parent sex	0.14	0.00	0.27	0.00	0.25	0.00
Time × K6-cat	10.25**	0.02	2.61	0.01	57.10**	0.11

*p < 0.05 **p < 0.01

### Parenting Team

The final set of models considered all outcomes in the context of the parenting team, that is, parents from two-parent families completing the program on their own versus with another caregiver. Preliminary analyses exploring associations between participation status and pre-intervention measures, indicated that participants who chose to complete on their own had lower levels of baseline SDQ Total Difficulties, *t*(261) = − 2.09, *p *< 0.05, and SDQ Conduct Problems, *t*(263) = − 2.59, *p *< 0.05, than those completing with their partner, suggesting that parents were more inclined to participate together when children had more behavioural and emotional problems. Consequently, SDQ-T-cat was included in the following models as a between-subject factor to control for differences in child behavioural and emotional problems across groups. Similar to the earlier models, all outcomes showed significant improvements over time (see Table 5). These results were not affected by parent team participation status (all interactions *p*’s> 0.05) showing that intervention effects were comparable among those completing on their own or with another caregiver. Parent team participation status showed only one significant main effect when parenting was the outcome variable, whereby parents choosing to complete the program on their own reported more negative parenting than those completing the program with their partner.

**Table 5:** Mixed-model parenting team results for child and parent outcomes

Variables	SDQ total difficulties		SDQ conduct problems		Parenting (PAFAS)		Interparental conflict (PPC)		Parental mental health (K6)	
	*F* (df)	η_p_^2^	*F* (df)	η_p_^2^	*F* (df)	η_p_^2^	*F* (df)	η_p_^2^	*F* (df)	η_p_^2^
Within-subject										
Time	134.13** (1,252)	0.35	102.97** (1,255)	0.29	117.95** (1,255)	0.32	44.18** (1,255)	0.15	15.05** (1,255)	0.06
Between-subject										
Participation status	2.02 (1,252)	0.01	0.06 (1,255)	0.00	5.21* (1,255)	0.02	2.88 (1,255)	0.01	3.09 (1,255)	0.01
SDQ-T-cat	221.91** (3,252)	0.73	71.99** (3,255)	0.46	13.85** (3,255)	0.14	3.28* (3,255)	0.04	8.42** (3,255)	0.09
Interactions										
Time × Participation status	1.86 (1,252)	0.01	2.61 (1,255)	0.01	2.63 (1,255)	0.01	0.60 (1,255)	0.00	2.81 (1,255)	0.01
Time × SDQ-T-cat	21.67** (3,252)	0.21	9.96** (3,255)	0.11	1.74 (3,255)	0.02	0.28 (3,255)	0.00	1.63 (3,255)	0.02
Participation status × SDQ-T-cat	3.40* (3,252)	0.04	1.87 (3,255)	0.02	2.28 (3,255)	0.03	1.07 (3,255)	0.01	0.21 (3,255)	0.00
Time × Participation status × SDQ-T-cat	0.60 (3,252)	0.01	0.07 (3,255)	0.00	1.01 (3,255)	0.01	2.20 (3,255)	0.03	1.88 (3,255)	0.02

*p < 0.05 **p < 0.01

### Benchmarking Analysis

Outcomes on SDQ Conduct Problems for a subsample of children rated *high* or *very high* were compared with children with similar SDQ conduct problem ratings who had been previously treated with the core parenting intervention via face–face or telehealth (benchmarking samples). Table 1 in supplementary information displays child adjustment and demographic data across the *ParentWorks* and benchmarking groups, and significant differences in child age, sex, and SDQ Total Difficulties were found. While Scheffe post hoc analyses did not indicate significant differences in child age between groups (M_diff_: *ParentWorks* vs. Face-face = − 0.77, *Scheffe p* > 0.05; *ParentWorks* vs. Telehealth = − 0.85, *Scheffe p* > 0.05), univariate *t*-tests indicated that children in the *ParentWorks* group were younger than those in the Telehealth group (*ParentWorks* vs. Face–face: *t*(232) = 1.92, *p* > 0.05; *ParentWorks* vs. Telehealth: *t*(236) =  − 2.18, *p* < 0.05). Scheffe post hoc analyses indicated that the *ParentWorks* group had lower SDQ Total Difficulties compared to the Telehealth group (M_diff_: *ParentWorks* vs. Face–face = − 1.46, *Scheffe p* > 0.05; *ParentWorks* vs. Telehealth = − 2.24, *Scheffe p* < 0.05). As such, child age and SDQ Total Difficulties were used as covariates in the benchmarking analysis to control for group differences that might potentially influence outcomes.

A repeated-measures ANOVA was used to test group differences on SDQ Conduct Problems at pre- and post-treatment (Table 2, Fig. 1 in supplementary information). Results indicated time effects whereby all treatment conditions were associated with significant pre- to post-treatment improvements in conduct problems. The ANOVA did not indicate significant effects for either sample-group or sample-group × time, after controlling for SDQ Total Difficulties and interactions between time × age and time × SDQ Total Difficulties.

### Cost Analysis

This section presents basic estimates of the program costs (i.e., costs associated with developing and delivering the intervention) and benefits from the intervention (measured in terms of cost savings for children who moved out of the clinical range of conduct problems following the program). The *ParentWorks* development and launch cost including website development, video production, and staffing was AU$389,595 (referred to as *sunk costs*). The ongoing costs for hosting, support and maintenance of *ParentWorks* are approximately AU$24,464 per annum. Based on 2014–2015 estimates [40], 97% of households with children aged under 15 years had access to the internet at home through a desktop/laptop computer, mobile/smart phone, or tablet. This means that computing facilities and Internet access required to complete the program are already available in almost all Australian households, and therefore, these costs were not included in the current analysis.

Based on previous literature [41, 42], the incremental costs (determined by subtracting the total costs of disorder from the costs of the non-problem group) of conduct disorder (CD) can be estimated at AU$213,825 for boys and AU$18,862 for girls (all values corrected for 2016 inflation rates; Reserve Bank of Australia). This includes costs associated with health, crime, government benefits, educational needs, foster and residential care, and interpersonal problems. We computed the proxy diagnosis of CD in the sample by using a cut-off point of 6 on the SDQ-CP scale, which indicates a likely CD diagnosis [43]. From pre- to post-intervention, 50 children (36 boys and 14 girls) moved out of the clinical range of likely CD diagnosis. Given the incremental costs of CD and based on the most severe cases, *ParentWorks* potentially saved AU$7,513,052, after subtracting the sunk costs for program development along with annual maintenance costs.

## Discussion

This study aimed to evaluate an entirely self-directed, universal online intervention designed to increase father engagement, reduce child behavioural problems and promote positive parenting. Findings provide empirical support for the use of *ParentWorks*. As expected, results showed significant reductions in child emotional/behavioural symptoms, conduct problems, dysfunctional parenting, interparental conflict, and parental mental health following the intervention. Average improvements were medium to large which is consistent with available literature on parenting programs [9, 44], and the strongest effect sizes were found for child outcomes which were the main target of the intervention. Furthermore, the current models showed that intervention effects on child outcomes were moderated by the initial severity of behavioural problems, as consistently reported in the literature [27]. This finding highlights that children with the most severe symptoms benefit most from universal interventions. However, contrary to some research suggesting that parenting programs are more effective for younger children [44], child age did not influence the outcomes of the program. This is likely because the current study largely included younger children; in fact, the majority (57%) of the sample was under age 6. It is also possible that child age does not affect parenting program outcomes and earlier interventions are not more effective, as shown in a recent meta-analysis [45].

In relation to parent outcomes, there were improvements in parenting, interparental conflict, and parental mental health after parents completed *ParentWorks*. Further, while parental mental health did not interact with improvements in interparental conflict, it did interact with improvements in parenting and parental mental health. Parents who were initially in the clinical range on mental health problems benefitted most from the program, in terms of change in parenting. This was likely to be due to their higher scores on dysfunctional parenting at the start of the program. Further, for those that reported high levels of child behaviour problems at pre-intervention, the online self-directed program produced similar outcomes to face-to-face and therapist-assisted online formats.

The *ParentWorks* program was unique in successfully recruiting a large number of fathers—36.6% of the total group completing the program (or 41.08% when excluding single mothers), which is double the rate previously reported in the literature [17]. This study examined program outcomes in the context of parent sex and the results showed it did not affect the intervention outcomes. Parent sex has rarely been considered a moderator in parenting programs largely because of the substantial focus of available literature on mothers [16]. Our study contributes an important finding that mothers and fathers seemed to benefit equally from the program. This study was also uniquely positioned to examine the effects of parenting team participation on intervention outcomes. Despite previous research showing that the involvement of fathers led to significantly improved intervention outcomes for child behaviour and parenting [19], the current study did not support the idea that participation of the parenting team is more beneficial than completing the program alone (where other caregivers are available). The participation status among married or de facto caregivers did not significantly affect any of the outcomes and results were comparable between those who completed the program on their own or with the other caregiver. Furthermore, all parents were highly satisfied with the program and no sex differences in satisfaction ratings were found. This is important as *ParentWorks* was designed to be father-friendly and appealing to both mothers and fathers [30]. Finally, the program can be considered cost-effective given substantial improvements in child and parent outcomes and potential savings due to decreased levels of child conduct problems and diagnoses. Despite substantial initial sunk costs associated with program development, the ongoing costs of program maintenance are minimal, and therefore *ParentWorks* could be expected to increase in cost-effectiveness if disseminated more widely.

This study has several strengths including a relatively large sample and the investigation of unique research questions regarding parent sex effects as well as parent participation status. The current study used a community-wide open-trial design that emphasised universal availability and recruitment into the intervention. More definitive conclusions about efficacy would have been aided by the inclusion of a control or wait-list group to control for child maturation or regression to the mean effects, as well as using more objective measures of child and parent outcomes, such as teacher rated measures and observational measures. Future studies should aim to conduct a randomised controlled trial of *ParentWorks* and other self-directed parenting interventions to control for a range of confounding factors. Future studies should also include follow-up to determine the maintenance of intervention effects over time. The cost analysis included in this study also assumed that the positive effects of the intervention were long-lasting, and more robust cost analyses taking into account these longer-term effects should be conducted. This study was entirely dependent on parent reports and the use of data from other informants or more objective assessment measures of child and parent outcomes would have reduced the risk of bias. However, this was not feasible due to the universal and online nature of the program. It is also important to note that only a small proportion of parents who registered completed the program and that the sample was highly educated (53% had university degrees, compared to average of 31% in Australia), which limits the generalisability of the findings. Given the high drop-out rates, it may be that the significant program effects were largely due to the self-selected nature of those who completed the program, and not representative of all parents in the population. It was not feasible to conduct intent-to-treat analysis in the present study, but this is important to conduct in future studies of self-directed parenting interventions.

With these caveats in mind, offering an entirely self-directed online program appears to be effective in reducing child behavioural problems, dysfunctional parenting, interparental conflict, and parental mental health. *ParentWorks* was designed as a universal prevention and intervention program accessible to all families, especially in rural and remote areas. The fact that it can be fully delivered online, without the support of trained practitioners, reduces the issue of accessibility that is often associated with parenting programs and considered a key barrier to help seeking [13].

## Summary

This study suggests that self-directed online programs could be an effective way of reducing child behavioural problems at the population level, given their potential to reach a large number of families. Substantial benefits can be achieved without practitioner support and at relatively low cost, which supports the universal use of the program as well as its use by more targeted samples, for example, parents on waiting lists for clinical services. Future studies should consider the long-term effects of the intervention and its delivery within a variety of healthcare contexts.

## Electronic supplementary material

Below is the link to the electronic supplementary material.

Supplementary material 1 (DOCX 40 kb)

## References

[1] Polanczyk GV (2015). Annual research review: a meta-analysis of the worldwide prevalence of mental disorders in children and adolescents. J Child Psychol Psychiatry.

[2] American Psychiatric Association (2013). Diagnostic and statistical manual of mental disorders, DSM-5.

[3] Brennan LM (2012). Longitudinal predictors of school-age academic achievement: unique contributions of toddler-age aggression, oppositionality, inattention, and hyperactivity. J Abnorm Child Psychol.

[4] Colman I (2009). Outcomes of conduct problems in adolescence: 40 year follow-up of national cohort. BMJ.

[5] Fergusson DM, Horwood J, Ridder EM (2005). Show me the child at seven: the consequences of conduct problems in childhood for psychosocial functioning in adulthood. J Child Psychol Psychiatry.

[6] Moilanen KL, Shaw DS, Maxwell KL (2010). Developmental cascades: externalizing, internalizing, and academic competence from middle childhood to early adolescence. Dev Psychopathol.

[7] Knapp M, Scott S, Davies J (1999). The cost of antisocial behaviour in younger children. Clin Child Psychol Psychiatry.

[8] Eyberg SM, Nelson MM, Boggs SR (2008). Evidence-based psychosocial treatments for children and adolescents with disruptive behavior. J Clin Child Adolesc Psychol.

[9] Kaminski JW, Claussen AH (2017). Evidence base update for psychosocial treatments for disruptive behaviors in children. J Clin Child Adolesc Psychol.

[10] Barlow J (2012). Group-based parent training programmes for improving parental psychosocial health. Cochrane Database Syst Rev.

[11] Furlong M (2012). Behavioural and cognitive-behavioural group-based parenting programmes for early-onset conduct problems in children aged 3 to 12 years. Cochrane Database Syst Rev.

[12] Kazdin AE, Blase SL (2011). Rebooting psychotherapy research and practice to reduce the burden of mental illness. Perspect Psychol Sci.

[13] Reardon T (2016). What do parents perceive are the barriers and facilitators to accessing psychological treatment for mental health problems in children and adolescents? A systematic review of qualitative and quantitative studies. Eur Child Adolesc Psychiatry.

[14] Baumel A (2016). Digital parent training for children with disruptive behaviors: systematic review and meta-analysis of randomized trials. J Child Adolesc Psychopharmacol.

[15] Nieuwboer CC, Fukkink RG, Hermanns JM (2013). Online programs as tools to improve parenting: a meta-analytic review. Child Youth Serv Rev.

[16] Panter-Brick C (2014). Practitioner review: engaging fathers-recommendations for a game change in parenting interventions based on a systematic review of the global evidence. J Child Psychol Psychiatry.

[17] Fletcher R, Freeman E, Matthey S (2011). The impact of behavioural parent training on fathers’ parenting: a meta-analysis of the Triple P-Positive Parenting Program. Father J Theory Res Pract Men Father.

[18] Huntington C, Vetere A (2015). Coparents and parenting programmes: do both parents need to attend?. J Fam Ther.

[19] Lundahl BW (2008). A meta-analysis of father involvement in parent training. Res Soc Work Pract.

[20] Frank TJ (2015). Using father preference data to increase father engagement in evidence-based parenting programs. J Child Fam Stud.

[21] Tully LA (2017). Optimizing child outcomes from parenting interventions: fathers’ experiences, preferences and barriers to participation. BMC Public Health.

[22] Dadds MR, Hawes DJ (2006). Integrated family intervention for child conduct problems: a behaviour-attachment-systems intervention for parents.

[23] Kirkman JJ, Hawes DJ, Dadds MR (2016). An open Trial for an E-health treatment for child behavior disorders II: outcomes and clinical implications. Evid-Based Pract Child Adolesc Ment Health.

[24] Dadds MR (2019). Therapist-assisted online treatment for child conduct problems in rural and urban families: two randomized controlled trials. J Consult Clin Psychol.

[25] Dadds MR (2018). Keeping parents involved: predicting attrition in a self-directed, online program for childhood conduct problems. J Clin Child Adolesc Psychol.

[26] Gardner F (2010). Who benefits and how does it work? Moderators and mediators of outcome in an effectiveness trial of a parenting intervention. J Clin Child Adolesc Psychol.

[27] Shelleby EC, Shaw DS (2014). Outcomes of parenting interventions for child conduct problems: a review of differential effectiveness. Child Psychiatry Hum Dev.

[28] Baydar N, Reid MJ, Webster-Stratton C (2003). The role of mental health factors and program engagement in the effectiveness of a preventive parenting program for Head Start mothers. Child Dev.

[29] Des Jarlais DC, Lyles C, Crepaz N (2004). Improving the reporting quality of nonrandomized evaluations of behavioral and public health interventions: the TREND statement. Am J Public Health.

[30] Tully LA (2017). Study protocol: evaluation of an online, father-inclusive, universal parenting intervention to reduce child externalising behaviours and improve parenting practices. BMC Psychol.

[31] Tully LA (2018). Evaluation of ‘The Father Effect’ media campaign to increase awareness of, and participation in, an online father-inclusive parenting program. Health Commun Inform.

[32] Goodman R (1997). The strengths and difficulties questionnaire: a research note. J Child Psychol Psychiatry Allied Discip.

[33] Furukawa TA (2003). The performance of the K6 and K10 screening scales for psychological distress in the Australian National Survey of Mental Health and Well-Being. Psychol Med.

[34] Kessler RC (2002). Short screening scales to monitor population prevalences and trends in non-specific psychological distress. Psychol Med.

[35] Sanders MR (2014). Parenting and Family Adjustment Scales (PAFAS): validation of a brief parent-report measure for use in assessment of parenting skills and family relationships. Child Psychiatry Hum Dev.

[36] Dadds MR, Powell MB (1991). The relationship of interparental conflict and global marital adjustment to aggression, anxiety, and immaturity in aggressive and nonclinic children. J Abnorm Child Psychol.

[37] Turner KM, Markie-Dadds C, Sanders MR (2010). Facilitator’s manual for group triple P.

[38] Eyberg SM, van de Creek L, Knapp S, Jackson T (1993). Consumer satisfaction measures for assessing parent training programs. Innovations in clinical practice: a source book.

[39] Goodman R (2015) Scoring the strengths & difficulties questionnaire. www.sdqinfo.com. Accessed 1 Sept 2016

[40] Australian Bureau of Statistics (2016) Household Use of Information Technology, Australia, 2014–15. http://www.abs.gov.au/AUSSTATS/abs@.nsf/Lookup/8146.0Main+Features12014-15?OpenDocument. Accessed 16 July 2018

[41] Mihalopoulos C (2007). Does the Triple P-Positive parenting program provide value for money?. Aust N Zeal J Psychiatry.

[42] Scott S (2001). Financial cost of social exclusion: follow up study of antisocial children into adulthood. Br Med J.

[43] Silva T, Osório FL, Loureiro SR (2015). SDQ: discriminative validity and diagnostic potential. Front Psychol.

[44] Sanders MR (2014). The Triple P-Positive Parenting Program: a systematic review and meta-analysis of a multi-level system of parenting support. Clin Psychol Rev.

[45] Gardner F (2019). The earlier the better? Individual participant data and traditional meta-analysis of age effects of parenting interventions. Child Dev.

