# Supplementary material for: ParentWorks: Evaluation of an Online, Father-Inclusive, Universal Parenting Intervention to Reduce Child Conduct Problems
Source: Child Psychiatry Hum Dev. 2019 Oct 24;51(4):503–13. doi: 10.1007/s10578-019-00934-0 (PMC7347669; doi:10.1007/s10578-019-00934-0)

Supplementary Table 1: Means and SDs comparing ParentWorks and Benchmarking sample on key adjustment and demographic variables.

|  | Face-face | Telehealth | ParentWorks† | Statistic |
| --- | --- | --- | --- | --- |
| *Demographics* |  |  |  |  |
| Age child M (SD) | 7.07 (2.31) | 7.15 (2.2) | 6.3 (3.24) | *F*(2,318) = 3.50* |
| Gender child N (%) |  |  |  | χ^2^(2) = 9.08* |
| Boys | 69 (83.1%) | 68 (78.2%) | 100 (66.2%) |  |
| Girls | 14 (16.9%) | 19 (21.8%) | 51 (33.8%) |  |
| Mother's education^1^ M (SD) | 3.18 (.68) | 3.21 (.77) | 3.39 (.79) | *F*(2,305) = 2.50 |
| Marital status (N) |  |  |  | χ^2^(2) = 5.93 |
| Married/Defacto | 29 (36.7%) | 19 (22.1%) | 55 (36.4%) |  |
| Single/Separated/Divorced | 50 (63.3%) | 67 (77.9%) | 96 (63.6%) |  |
| *SDQ Scales* |  |  |  |  |
| Conduct | 5.43 (1.69) | 5.8 (1.98) | 5.64 (1.64) | *F*(2,297) = .96 |
| Total | 20.87 (4.82) | 21.65 (5.44) | 19.41 (6.08) | *F*(2,296) = 4.50* |

*Note*. ^1^ Mothers education was entered as continuous due to insufficient number of participants in cells to calculate chi-square statistic; †ParentWorks sample consists of families with children rated *high* or *very high* for conduct problems; * *p*-value < .05.

Supplementary Table 2. Repeated measures ANOVA comparing outcomes on SDQ conduct problems (mother’s report) for ParentWorks and benchmarking groups.

|  | SDQ Conduct Problems | |
| --- | --- | --- |
| Variables | F(df) | Partial eta squared |
| *Within-subject* |  |  |
| Time | 5.03 (1,259)* | .02 |
| *Between-subject* |  |  |
| Sample-group | .16 (2,259) | .00 |
| *Interactions* |  |  |
| Time x Sample-group | 2.25 (2,259) | .02 |
| *Covariate* |  |  |
| Age | .31 (1,259) | .00 |
| SDQ Total Difficulties | 89.77 (1,259)* | .26 |
| Time x Age | 4.18 (1,259)* | .02 |
| Time x SDQ Total Difficulties | 4.6 (1,259)* | .02 |

*p<.05 **p<.01

Supplementary Figure 1: Outcomes at pre- and post-intervention on SDQ conduct problems for ParentWorks and Benchmarking groups.


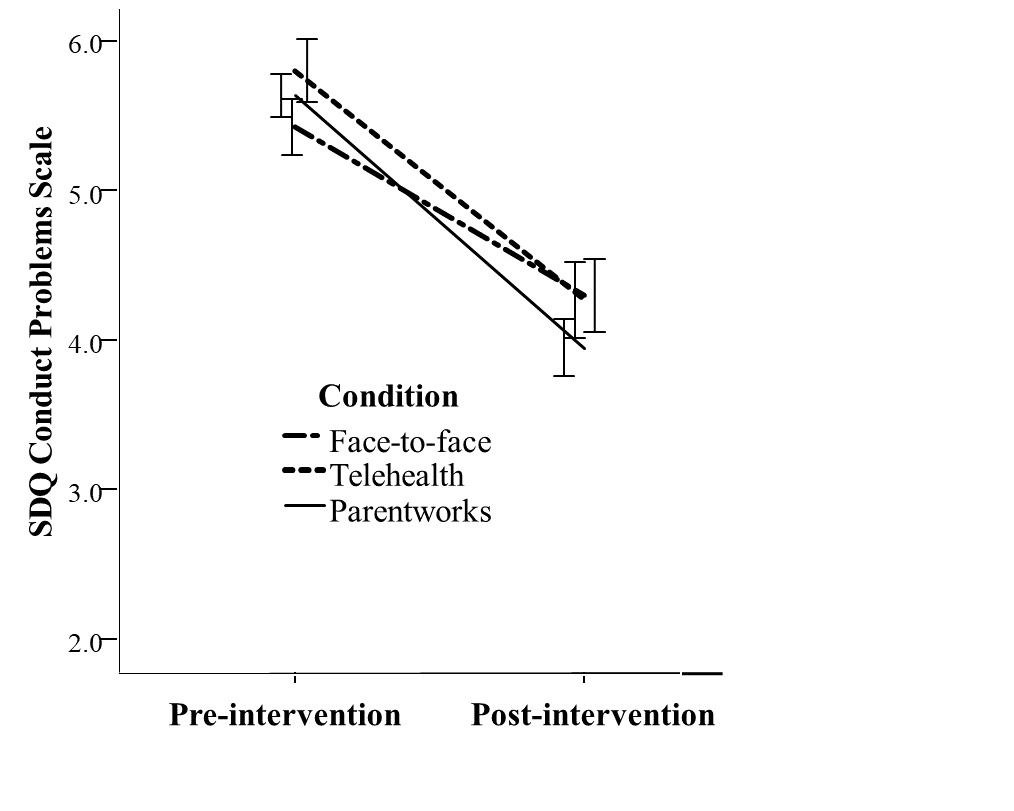

Supplement: Supplementary file 1 — Supplementary material 1 (DOCX 40 kb) [file 10578_2019_934_MOESM1_ESM.docx]
